# Auto Visual AFP Detection and Response (AVADAR) Improved Polio Surveillance in Lake Chad Polio Outbreak Priority Districts

**DOI:** 10.29245/2578-3009/2021/S2.1101

**Published:** 2021-04-13

**Authors:** Mamadou Diallo, Alimou Traore, Myk Mwanza Nzioki, Ayangma Richelot, Kouryana Stephane, Joseph Okeibunor, Mkanda Pascal, Samuel Okiror, Johnson Ticha

**Affiliations:** 1 WHO Regional Office for Africa (WHO AFRO), Brazzaville, Congo; 2 WHO Headquarters, Geneva; 3 WHO Chad; 4 WHO Horn of Africa Coordination Office (HOA), Nairobi KENYA

**Keywords:** AFP, AVADAR, Poliovirus, Outbreak, Lake Chad Polio Outbreak Priority Districts

## Abstract

The Auto Visual AFP Detection and Response (AVADAR) is a community-based digital platform that deals with the collection and distribution of real-time information. AVADAR makes it possible to report suspected cases of paralysis in the field at the central level.

Once a suspected Acute Flaccid Paralysis (AFP) case is detected, a series of reports are sent to the following stakeholders: the nearest training officer, the district focal point, the district AVADAR team, the regional focal point, the central level of the Ministry of Health (MoH) and World Health Organization Country Office (WCO) by SMS and email. The health worker will go to the field to join the community informant who notified the case for a clinical investigation. At the end of this investigation, the health worker via a smartphone will submit an investigation report validating or invalidating the suspected case notified as a true case of AFP or False case.

A small server called a gateway is positioned at the central level to ensure the information link between community informants and health workers in each district.

A large server is placed in Geneva at Novel-T which allows all countries to connect and view the data in real time.

The geolocation of all alerts and investigations of AFP cases is the cornerstone of AVADAR data

## Avadar Sructure

## Method

Following the demonstrated effectiveness of the Auto Visual AFP Detection and Response (AVADAR) app to facilitate more accurate and timely report of AFP cases, in a small pilot study, it was scaled up for use in the Lake Chad polio priority districts. This was implemented in 25 priority districts, following a systematic process. A series of cascade training was conducted after the selection of community informants.

The results show that AVADAR improved surveillance in the Lake Chad polio priority districts. The number of AFP true cases reported through the AVADAR technology far outstripped those from the traditional case finding system. The trend of AFP case reporting was also on the rise. In 2017, only four cases were reported. But with the introduction of AVADAR in 2018 there were 35 true AFP cases reported in the priority districts. AVADAR also made it possible to report AFP cases from Island settlements that were previously not reported on.

## Introduction (Surveillance)

Surveillance for acute flaccid paralysis (AFP) among children aged <15 years with testing of stool specimens for WPV and vaccine-derived polioviruses (VDPVs) in World Health Organization (WHO)–accredited laboratories of the Global Polio Laboratory Network (GPLN) is a principal means of detecting poliovirus^1–3^. The sensitivity of AFP surveillance is critical for prompt detection and interruption of polio virus transmission. Two performance indicators for assessing the quality of AFP surveillance are the non-polio AFP (NPAFP) rate and adequacy of stool specimen. The NPAFP rate is measured by the number of NPAFP cases per 100,000 children aged <15 years per year. An NPAFP rate ≥2 is considered sufficiently sensitive to detect circulating poliovirus. On the other hand, stool adequacy refers to two stool specimens collected ≥24 hours apart and within 14 days of paralysis onset and arrival at a WHO-accredited laboratory by reverse cold chain and in good condition, without leakage or desiccation from ≥80% of persons with AFP, which ensures sensitivity and provides the specificity to track poliovirus circulation^2^.

In 2016, an ongoing outbreak of type 1 wild poliovirus (WPV1) was detected in Nigeria. This was exactly two years after the last case in Nigeria, and indeed in the African Region^4,5^. That was followed by four cases of WPV1 have been reported in Borno State, Nigeria. This was a devastating shock and sudden end to the build up to celebrate polio free African Region. More importantly, it was a sad reality of the weakness in the AFP surveillance that was conducted in the region, especially in the Lake Basin area, encompassing the northern part of Nigeria with very high risk for polio transmission, where conflict and poor geographical terrain made access to many communities impossible. The other countries with high risk countries within this zone included Cameroon, Central African Republic, Chad and Niger.

In response, these countries of the Lake Chad subregion, declared the new outbreaks in Nigeria a regional public health emergency, with a regional outbreak response, coordinated with neighbouring countries. Given the realization of the surveillance gaps as the principal driver of these new outbreak, steps were taken to improve AFP surveillance activities. The Lake Chad Task Team for polio eradication deployed a number of innovative technologies such the use of innovative technologies such as ISS, Auto Visual AFP Detection and Response (AVADAR), eSurv and Geographical Information System (GIS), to improve detection and response to polio outbreaks. These innovations are now increasingly used by public health professionals to visualize and explore disease patterns^6–10^. Similar tools were also used in India to monitor population migration through railroads, and Google Earth was used to track the poliovirus down the Congo River^6^. More importantly, the Lake Chad polio outbreak response task team recorded dramatic success in interrupting the transmission of WPV1 outbreak in Nigeria as well as ensuring now new outbreak of WPV1 in the Lake Chad Outbreak Sub Region. This paper documents the deployment of the AVADAR to improve sensitivity of AFP surveillance in the Lake Chad Outbreak Priority Districts.

## AVADAR – An Innovation to Improve AFP Surveillance

Sequel to the report of an ongoing outbreak of wild poliovirus in northeast Nigeria in 2016, eHealth Africa (eHA) partnered with the Bill and Melinda Gates Foundation, the World Health Organization (WHO), and Novel-T to pilot a mobile-based surveillance app for Acute Flaccid Paralysis (AFP) in children called Auto-Visual AFP Detection and Reporting (AVADAR) app^11^. The new App was operationalized in this context and found to detect true cases of AFP, for the purpose of identifying and responding to potential cases of polio. The AVADAR app has a 30-second video of a child with AFP symptoms embedded in it which reminds Healthcare Workers and Community Informants to report suspected cases weekly. eHA deployed Android phones equipped with the AVADAR app to Community Informants in target regions with high risk of polio transmission in Nigeria.

A test run of in Nigeria showed that AVADAR could be used to improve AFP reporting even in areas with particular challenges like insecurity and limited geographical accessibility for surveillance activities. In the pilot local government area (LGA) in Nigeria where the project was first launched, 26 AFP cases were reported within the first 8 weeks as against 23 cases that were reported within 33 weeks before the pilot study was implemented. Furthermore, the geographical areas from where the AFP cases were reported spanned a wider area than what have been the source areas for AFP cases over the previous years. Besides this, some AFP cases that were not picked up by the traditional system were also detected and reported, albeit after 60 days of onset of paralysis. Nevertheless, perhaps one of the most important features of the AVADAR system is its ability to ensure that zero reporting takes place across a wider area. Thus, AVADAR’s implementation greatly increases the number of persons conducting active surveillance activities in the project area as well as attracting the attention of host government and partners to ensure surveillance quality improves. AVADAR was thus recommended by the GPEI partners to be scaled up to the Lake Chad Basin countries.

AVADAR was designed to strengthen AFP surveillance where there were perceived gaps or where there exist challenges or when there is the risk of missing AFP cases. The successful deployment of AVADAR had the following assumptions

- High level advocacy with the MoH to get their buy in to the ownership, support and guidance
- Functional health system to guide in the identification of point of entry of AVADAR. The AVADAR system uses various types of community informants and health personnel working in health facilities
- Mobile Network coverage prerequisite for AVADAR to work
- A network of volunteer community-based informants that consists of Community Citizen, Bone setters, Traditional healers, Religious leaders, Traditional leaders, youth leaders, etc. who are well respected.

## Target Areas in Lake Chad Basin

**Figure 2:**
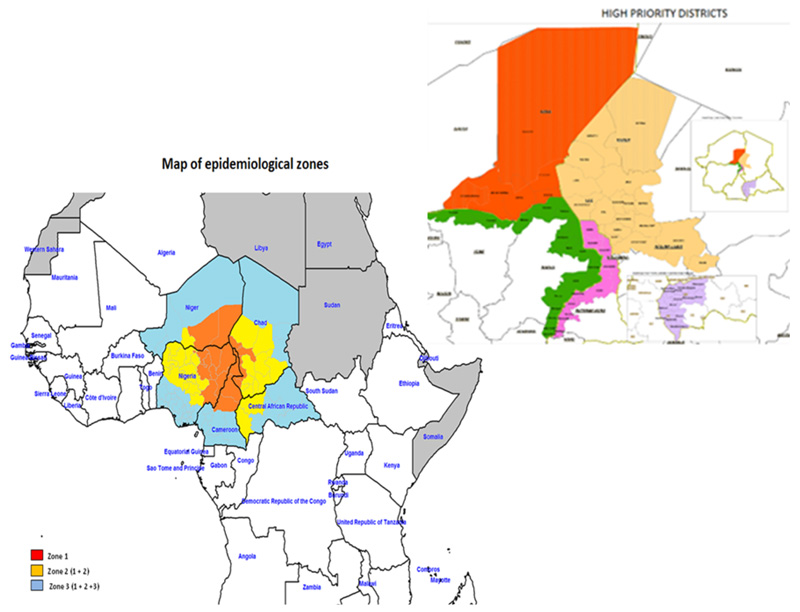
Showing the epidemiological zones with the priority districts marked for implementation of AVADAR

It will be recalled that on 10 August 2016, WHO received notification through the Global Polio Laboratory Network (GPLN) of the detection of 2 wild poliovirus type 1 (WPV1) cases in 2 LGAs of Borno, Nigeria: in Gwoza, one AFP case and 3 healthy contacts positive with onset of paralysis on 13 July 2016; and in Jere one healthy contact positive with onset of paralysis of index AFP case on 6 July 2016. The VP1 sequencing results indicate that Gwoza index case is a WPV1 with 41 nt. difference related to WEAFB1 genotype in cluster N7B. Closest matching viruses are Bama and Damaturu LGAs viruses of 2011.These isolates are the first to be detected in Nigeria since July 2014 and signify undetected WPV1 transmission for about five years in Nigeria and/or neighbouring countries in the Lake Chad basin (Cameroon, Niger, Chad and CAR). These viruses reported in Borno constitute a risk of spread within the country and to neighbouring Lake Chad basin countries. See Figure 2 below.

The outbreak response in the Lake Chad region was initially based on a multi-country regional response. Based on the context, epidemiology and the risks of spread, the areas were categorised into 3 epidemiological zones, namely:

– **Zone 1:** The Outbreak zone including areas in:
  ○ Cameroon: Extreme Nord, Nord, Adamawa
  ○ Chad: Lac, Hadjer Lamis, N’Djamena, Mayo Kebbi Est, Mayo Kebbi Ouest
  ○ Niger: Diffa and Zinder
  ○ Nigeria: Borno, Gombe, Adamawa, Taraba, Yobe
– **Zone 2:** Zone 1 + the neighbouring areas/countries:
  ○ Cameroon: Extreme Nord, Nord, Adamawa, Nord Ouest, Ouest
  ○ CAR: RS 2 and RS 3
  ○ Chad: Lac, Hadjer Lamis, N’Djamena, Mayo Kebbi Est, Mayo Kebbi Ouest, Kanem, Salamat, Batha, Guera, Tandjile, Logone Occidental, Logone Oriental, Chari Baguirmi, Mandoul, Moyen Chari
  ○ Niger: Diffa, Zinder and Maradi
  ○ Nigeria: Borno, Gombe, Adamawa, Tabara, Yobe, Jigawa, Bauchi, Kano, Katsina, Zamfara, Sokoto, Kebbi, Niger, Kaduna, FCT/Abuja, Nasarawa, Plateau, Benue
– **Zone 3:** Zone 1 + Zone 2 + the rest of states/regions of all 5 countries

The Lake Chad Basin consisted of five countries, namely Cameroon, Central Africa Republic (CAR), Chad, Niger and Nigeria. For the purpose of the Lake Chad Polio Outbreak response, a total of 62 priority districts were identified in these countries for the operation of the Task Team of the Lake Chad Basin Coordination based in Chad. These were 10 in Cameroon, 17 in Chad and 15 in Nigeria. CAR had 14 while the remaining six were in Niger.

## Process

The implementation of the AVADAR project in the Lake Chad Polio Outbreak Response districts involved different levels of personnel as well as activities like training of trainers (ToT), deployment and supervision of personnel, and monitoring and evaluation. There was thus a cascade of training of all the stakeholders (See Figure 3 below).

## Regional Epidemiologist

This is the head of the disease control section or department in the Ministry of health. AVADAR system will have to operate under his leadership and thus he/she is a key player. The regional epidemiologist will have the following tasks/activities:

○ Advocate for AVADAR ownership by Director of Disease Control/Director public health and other Central technical and administrative persons (Minister, Regional delegate of health etc).
○ Participate in the selection of the areas with surveillance gaps (evidenced by data and reports)
○ Lead in the selection of informants
○ Participate in the training of informants
○ Participate in the supervision/monitoring of informants once deployed in the filed
○ Weekly reporting on activities of AVADAR and on its impact on surveillance system.
○ He/she will also be receiving messages on AFP cases detected and ensure timely investigation.

## District Level Medical Officers

This is an important class of stakeholders because he/she is the one to lead the investigation of all suspected cases upon receipt of each alert (text message).

The surveillance officers have the following key tasks/functions to play:

○ Ensure all suspected cases are investigated within 48 hours
○ Supervise the informants (and their supervisors)
○ Review and provide solution to problems faced by informant
○ Ensure smart phones are functional
○ Participate in monthly meetings with informants
○ Maps of Alerts and investigation

## Health Workers

In the best case scenario, all health workers (surveillance focal persons in health facilities inclusive) in both private and public health facilities in the areas earmarked for deployment of AVADAR will be brought into the network. The head of a health facility (if not a surveillance focal person) would ideally be the person to manage the phone in collaboration with other health workers in the facility. In all designated facilities, an assistant Health Worker should be identified to provide the necessary support when the focal person is not available. The ideal situation is to have the focal person in each of the selected health facilities to be part of the system.

## Community Informants

Using established/existing surveillance Community informants’ network in the areas, the following individuals are generally identified in the community as informants:

○ Patent Medicine Vendors,
○ Traditional Birth Attendants,
○ Youth leaders,
○ Retired teachers,
○ Traditional healers
○ Bone setters
○ Faith-based healers (Religious leaders).

These individuals are well known in their communities and are potential persons to whom cases of AFP could be referred or reported to. So if a case of AFP occurs, they are one of the first people to see the case or to be informed of. Apart from waiting for cases, these Community informants are expected to do active case search by doing house to house visits during which they show the video of an AFP case to community members and ask them to report any such cases to them. Each informant is expected to visit 50 households within the first two weeks of being engaged. Subsequently, it is expected that he will visit these same households, at least once every month to remind household heads and their wives about AFP occurrence.

Where these informants have not been formally identified (names, location, address, phone numbers etc) it has to be done before AVADAR is initiated. Informants to serve in the AVADAR system are expected to be those who can read and write and who could be taught to use a smart phone or they are able to manipulate a smart phone already.

The regional and district teams underwent training of trainers (ToT) at the National level which was facilitated by WHO-AFRO and consequently conducted district level training in each district bringing together all the health workers for hands-on practical sessions.

Each of the health worker proceeded to train the identified community informants. In total 3,547 community informants received phones and monthly credit to purchase internet bundles. See Table 1 for distribution of community informants and phones within the Lake Chad Polio Outbreak Response.

Table 1: Distribution of personnel and mobile phones in the Lake Chad polio outbreak AVADAR project

## Monitoring Methods

Monitoring of the AVADAR project is one of the basic components of the project as monitoring will provide the information that will be useful in analyzing the implementation of the project, determining whether the inputs in the project are well utilized and Identifying challenges facing the project and finding timely solutions.

- **Using the dashboard on the server**

All alerts and investigations are recorded on the server. There is a provision made for basic analysis and reports for use by the supervisors at district, region and national levels

The dashboard provides information on the following:

○ Systemic and operational indicators
○ Functionality of the Gateways and server
○ Correct use of the application by the informants
○ The process of AFP case reporting and zero reporting.
○ Informant details of “Silent informants” or those informants that have sent incomplete location information or no location informant at all

### ARGUS section

*https://avadar.afro.who.lnt/sesDashboard/web*

### AVADAR section (add-on to ARGUS)

*https://avadar.afro.who.lnt/Dashboard*

- **Supervisory/field visits by national and state consultants**

The National consultants - BMGF and WHO consultants will be conducting a well-planned supportive supervision to Region Medical Officer, District Medical Officer, health Workers and informants using AVADAR checklist, to ensure the end users understand the AVADAR operations, the phones are being used as per guidelines, issue of troubleshooting and also to understand local challenges that end users may encounter.

- **Monthly review meetings with AVADAR informants**

At least one review meeting in each of the district Headquarters. Members of AVADAR national team with support from the WHO hub coordinator and Regional Medical Officer will coordinate the meetings. The meetingwill allow end users to share experiences, share data and receive feedbacks.

## Implementation

**Figure 4:**
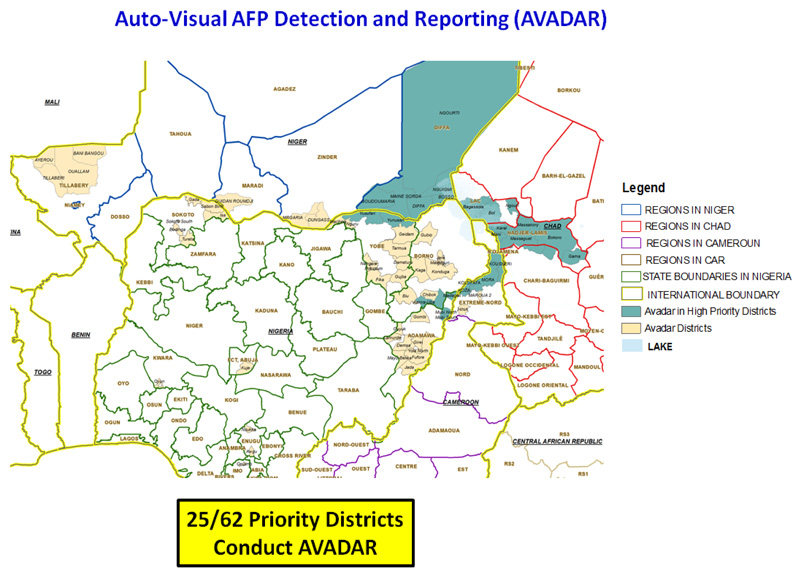
AVADAR conducted in high priority districts in the Lake Chad Basin

Figure 4 shows the priority districts where AVADAR was successfully deployed and implemented within the Lake Chad Polio Outbreak Response area. It shows the implementation took place in 25 of the 62 priority districts distributed across Cameroon, Chad, Niger and Nigeria. CAR could not conduct the project due political crises.

In Nigeria, implementation started with 4 states with two later included. There was a total of 42 local government areas. Implementation was possible even in security compromised areas. A total of 3290 informants were enrolled and selection of AVADAR areas are based on AFP Risk Assessment Matrix. The Matrix included 6 surveillance indicators, namely number of AFP reported; AFP Detection Rate; percentage of stool adequacy and percentage of silent wards, among others.

In Chad, the AVADAR project was effectively implemented in 441 settlements and a total of 104 special informants. The different immunization partners and stakeholders were actively involved. Cascaded training was conducted at different levels. Similarly, in Niger, there was a training of 9 EDC members from the 3 new districts. The training involved community leaders and relays in the health districts. Monthly monitoring meetings were conducted. Elaboration of the action Plan for the extension of AVADAR surveillance in the border districts was conducted. Similar implementation realities were recorded in Cameroon.

## Results

Table 2 shows the results of the implementation of the AVADAR project in the Lake Chad Polio high risk districts. Between 2018 and week 14 of 2019 a total of 20019 alerts were received and recorded. Eighteen thousand, eight hundred and thirty (94%) of these were investigated. Six hundred and thirty-four were confirmed true AFP cases. More sixty percent (61.4%) of the true AFP cases resulted from the use of AVADAR.

This trend was repeated in all the countries implementing AVADAR. In Cameroon where 105 (89%) of the 2715 investigated alerts were confirmed true AFP cases, more than two-third (72) were from AVADAR. Similarly, in Nigeria AVADAR picked up 72 true AFP cases compared to the 33 cases from the traditional surveillance process.

**Figure 5:**
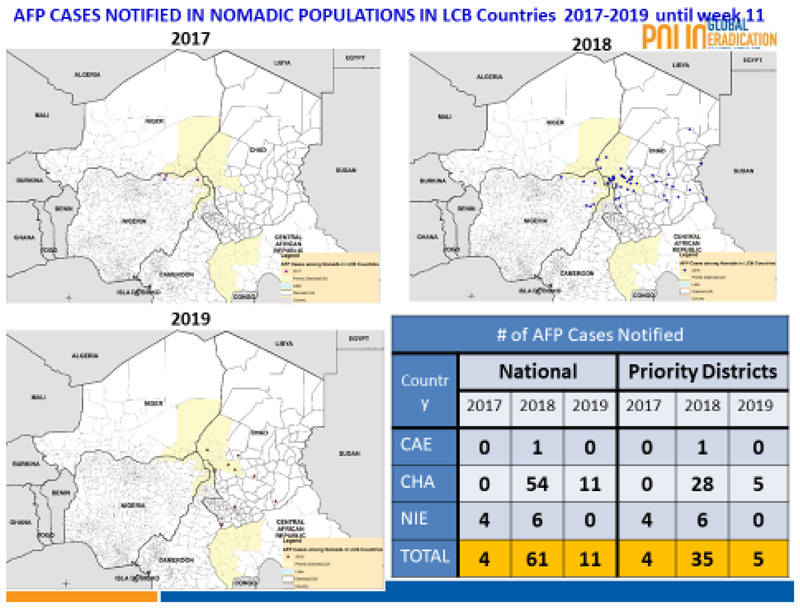
Trends in AFP cases reported in the Lake Chad Polio Priority Districts

Furthermore, there was a general improvement in the number of AFP cases report. Figure 5 shows that in the whole of 2017 four AFP cases were reported from the priority districts in Cameroon, Chad and Nigeria. With the implementation of AVADAR this improved, and 35 cases were picked up and within the first half of 2019 5 cases were picked up. The change was more dramatic in Chad where no AFP case was picked in 2017, but with the aid of AVADAR 28 cases were picked in 2018 and 5 within the first half of 2019.

With the AVADAR technology, it was now possible to pick and report AFP cases in hard to reach Island settlements. Figure 5 shows that between 2018 and week 11 of 2019, a total of 29 AFP cases were reported from Borno State, Nigeria (11) and Chad (18) with their precise geocodes recorded.

**Figure 7:**
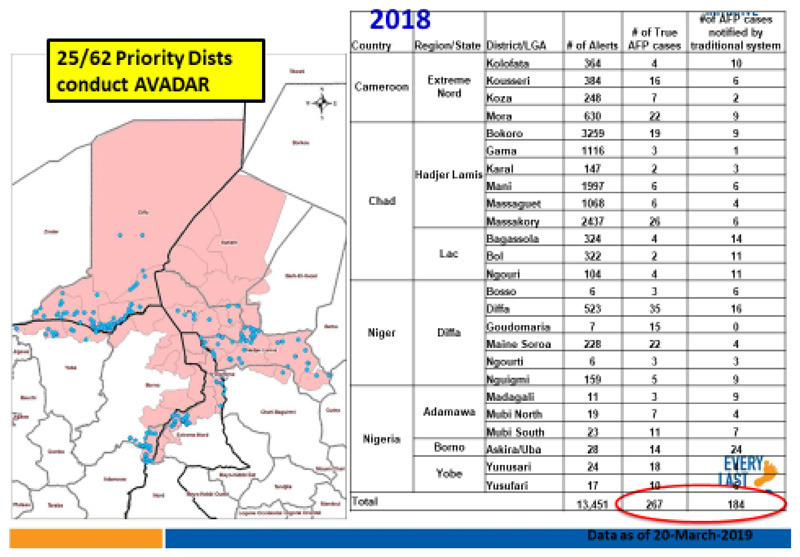
AVADAR True AFP Cases with Geocodes in Priority Districts, 2018

Figure 7 revealed that AVADAR was able to produce true AFP cases with geocodes in the priority districts. In 2018, of the total of 13,451 alerts from the four implementing countries of Cameroon, Chad, Niger and Nigeria, 267 were true AFP contributed by AVADAR compared to 184 from the traditional surveillance techniques.

**Figure 8:**
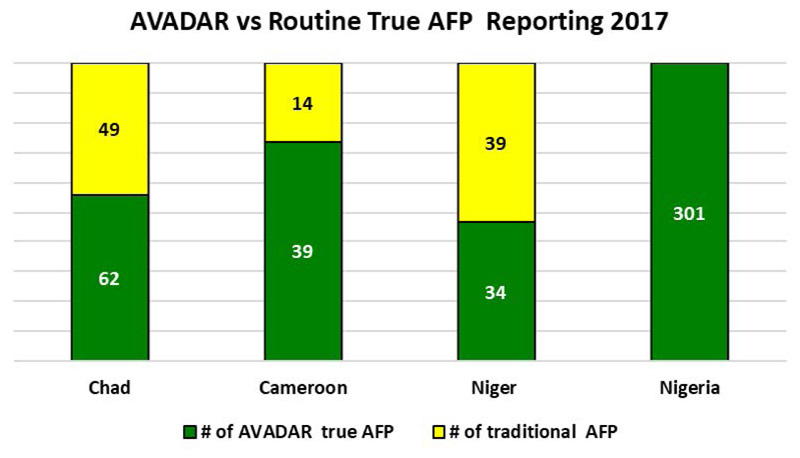
AFP cases notified via AVADAR vs traditional system in 2017

Over the years, we can clearly see that AVADAR has provided some support in terms of AFP detection in the Lake Chad Basin countries by the notification of AFP cases being consistently higher for AVADAR than the traditional system (see Figure 8 for 2017, Figure 9 for 2018 W1-W43 and Figure 10 for 2019 W1-W45).

**Figure 9:**
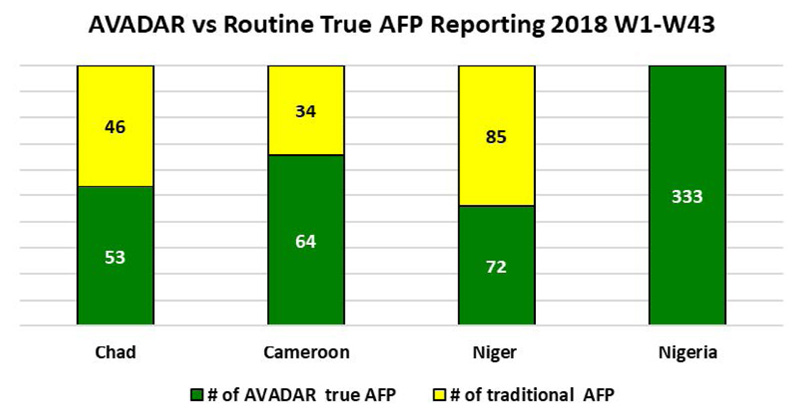
AFP cases notified via AVADAR vs traditional system in 2018 W1-W43

**Figure 10:**
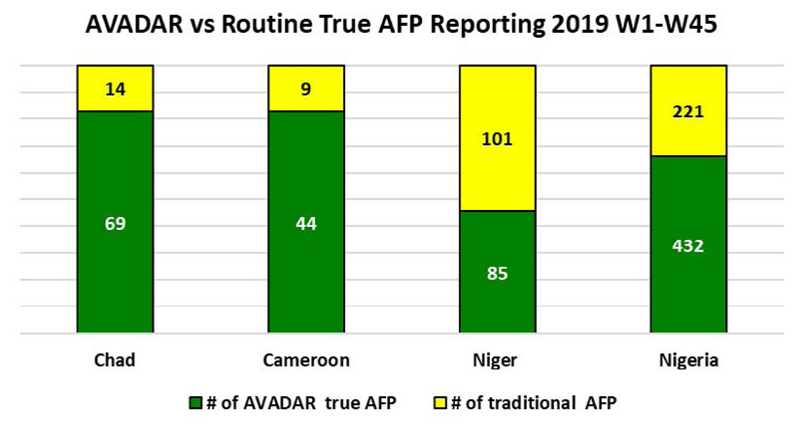
AFP cases notified via AVADAR vs traditional system in 2019 W1-W45

Note: Niger has more traditional cases because due of the presence of several NGOs which finance (by payment) community informants at each discovery of cases of AFP contrary to AVADAR which is done in the form of volunteer work Figure 9 10

## Discussion

The number of WPV cases reported in the Lake Chad polio priority districts declined to a point where is it possible to begin to consider the closure of the Task Team coordinating office in Chad. However, reported cVDPV cases have increased recently. Surveillance quality indicators improved in in the polio priority districts with the introduction of AVADAR. Unlike, in the past, it is not possible to make accurate report of cases with geocodes in previous inaccessible settlements, particularly in the Islands in Chad and those in parts of Northern Nigeria.

Strategies to strengthen AFP surveillance in areas where conflict occurs have included increased AFP case searches among camps for internally displaced persons, engagement of community members in inaccessible areas, and active case searches in newly accessible areas^12,13^. While one may argue that conflict might limit access to standard health facility–based surveillance, it is also true that community-based surveillance has demonstrated some levels of effectiveness in finding AFP cases, thus reducing the risk of poliovirus circulation in critical areas. This is very true of Somalia, where community volunteers have been instrumental in reporting AFP cases in inaccessible and partially accessible areas^14^. With the help of innovative applications like the AVADAR this will even be more facilitated as has been demonstrated in this project. The results here have shown AFP cases reported from Island settlements where such reports with previously impossible.

The extension has been done in the lake Chad districts rather than other districts with poor performance because of the proximity with task team base in Ndjamena and their financial support. The good performance on investigation by Chad is explain by money for fuel give to investigators, some remittance gives to them for each alert investigate, consultant participation in case validation. In the district with no case in routine surveillance, the activity is ongoing, but the high number of CI deployed on field is helping to detect case rapidly. For the maintenance of phones, the district coordinator of AVADAR have been trained by the eHealth during hand over process. Specials informants are still helpful particularly in area with low level of education.

For Data management, alerts situation is shared two time per week. AFRO and Novel T should look at the situation of missed alerts after division of district in two parts. Country is invited to develop the mechanism to face the progressive redraw of partners’ finance.

AVADAR has displayed its efficiency in the detections of AFP cases where the traditional might have missed those detections. AVADAR should only be considered complementary to the already existing traditional system of AFP detection and not a separate system altogether.

## Figures and Tables

**Figure 1:**
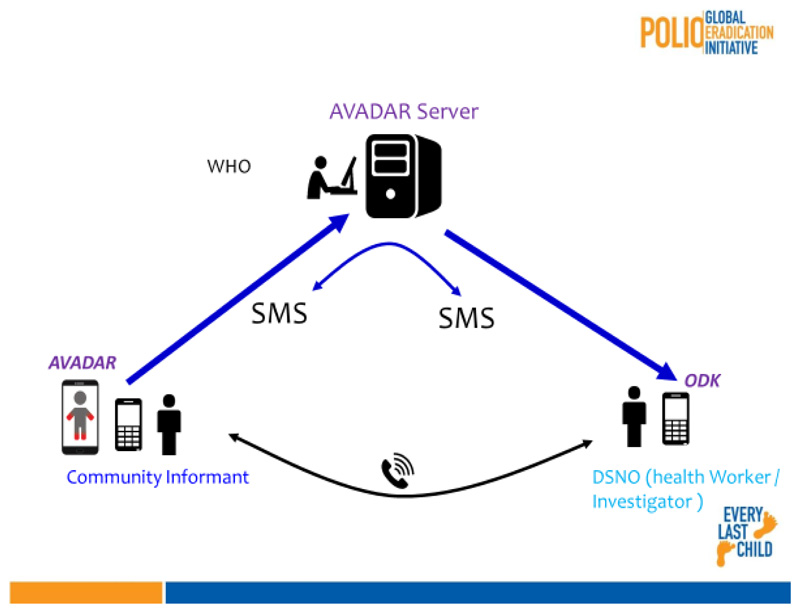
Overview of AVADAR infrastructure

**Figure 3:**
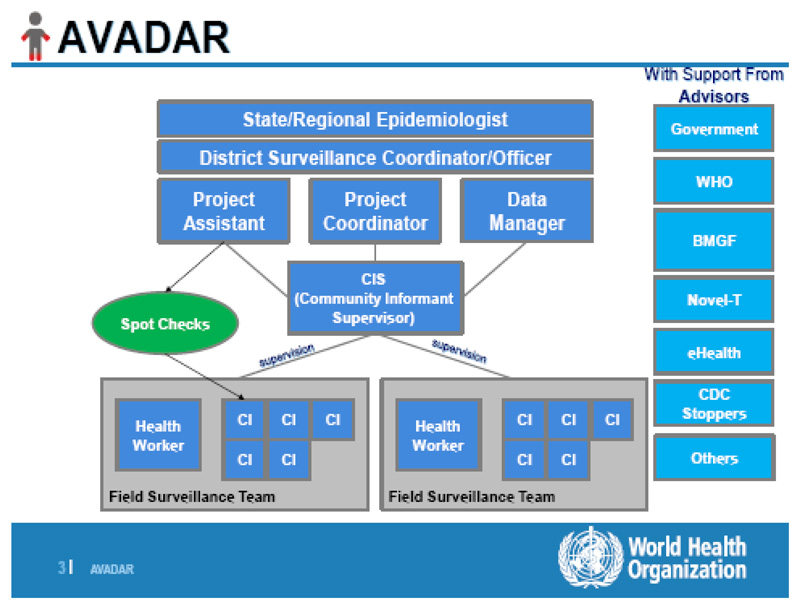
Stakeholders in Lake Chad Polio Outbreak Response AVADAR Project

**Figure 3A:**
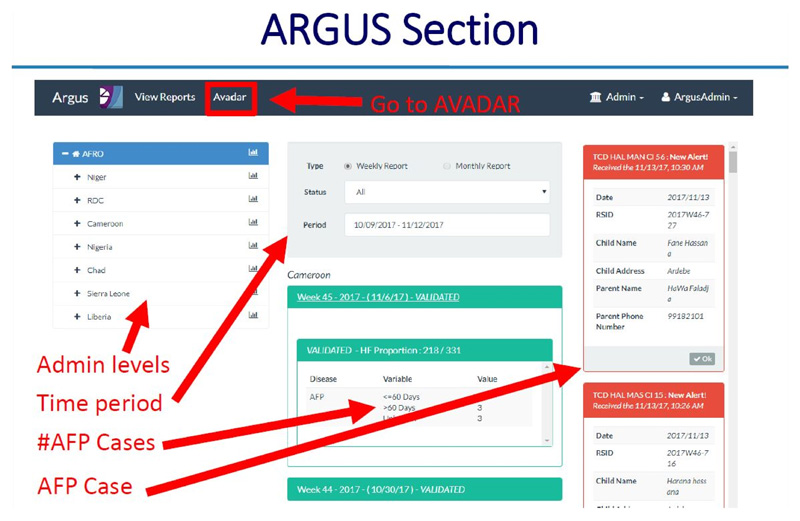


**Figure 3B:**
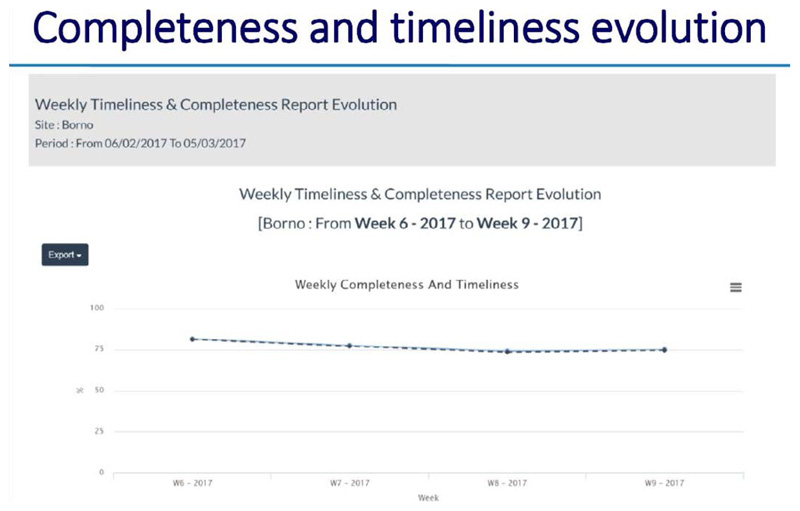


**Figure 3C:**
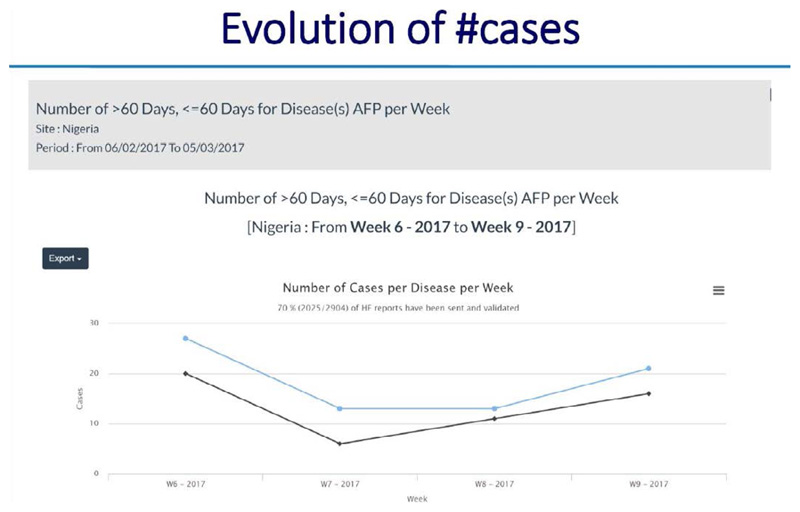


**Figure 3D:**
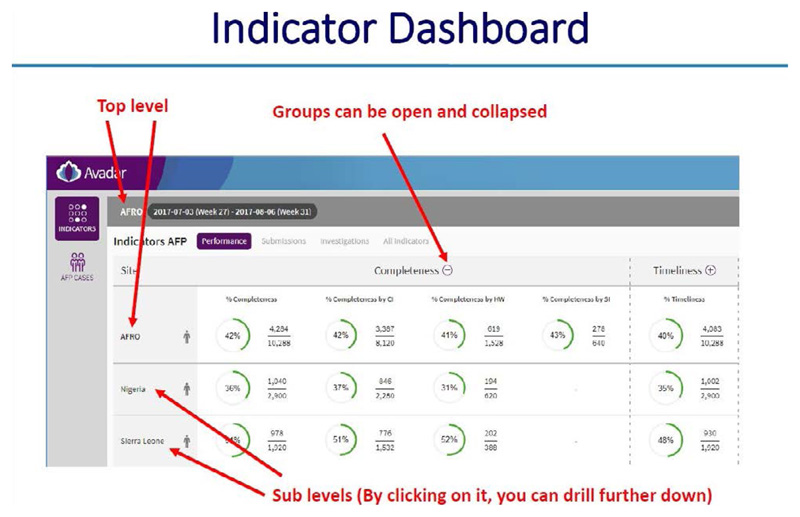


**Figure 3E:**
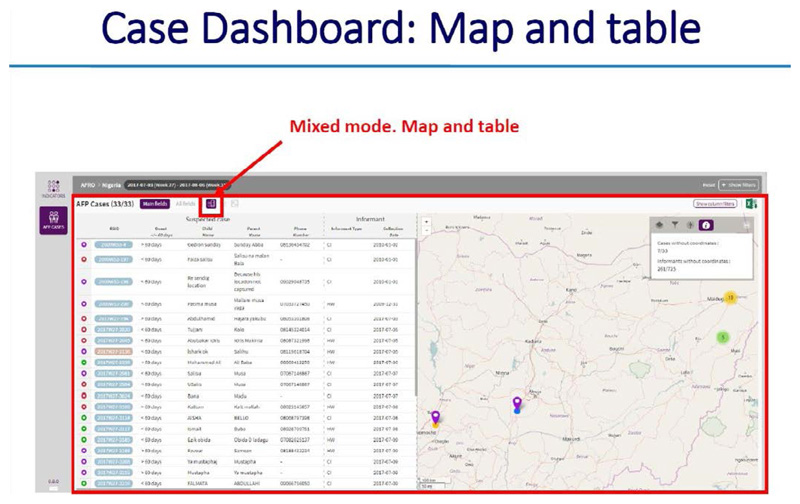


**Figure 3F:**
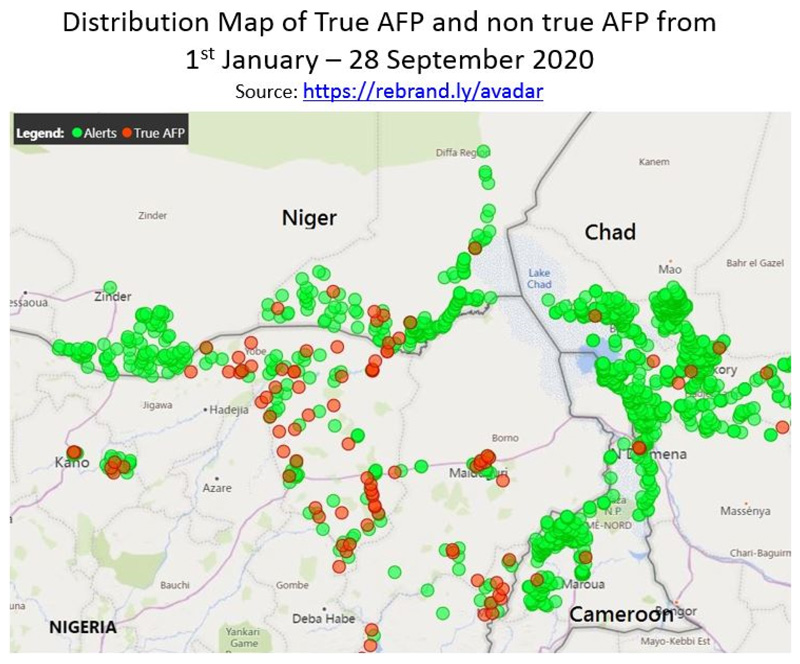


**Figure 6:**
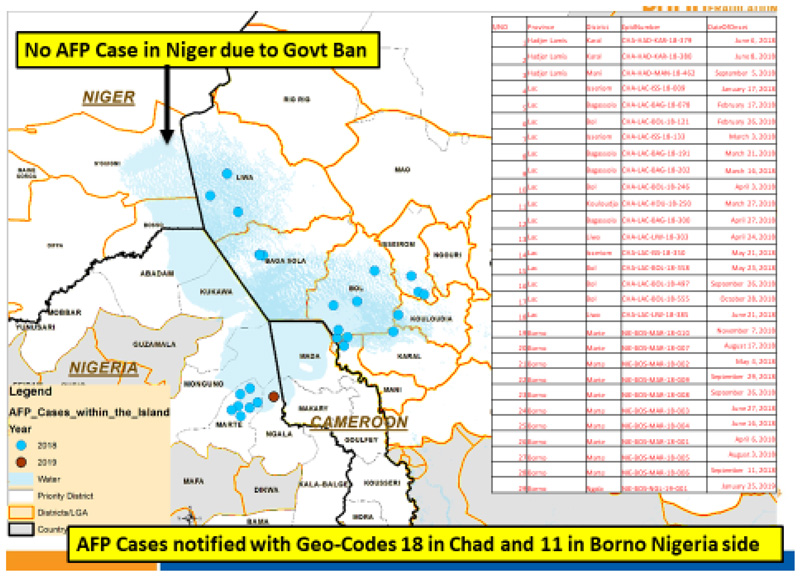
AFP cases notified with Geo-Codes 18 in Chad and 11 in Borno, Nigeria (2018-Week 11, 2019)

**Table 1:** Training and deployment in Chad

Country	Type of implementation	Region/State	Number of Districts/LGAs	Investigators	Health Workers	Community Informants	Mobile Phones
Cameroon	Pilots	Far North	3	90	15	335	700
	Expansion	Far North	3	72		308	550
Chad	Pilots	Hadjer Lamis	6	98	67	334	600
	Expansion	Lac	3	57		225	805
Niger	Pilots	Diffa	3	103		273	700
	Expansion	Maradi/Zinder	3	75			605
Nigeria	Pilots 2016	Abuja/Kwara	2	7	58	140	250
	Scale up 2016	Borno	8	25	97	430	663
	Epansion2 2017	Sokoto/Adamawa/Yobe	31	65	821	1,502	2870
	Expansio3 2019	NA	NA	NA	NA	NA	NA
Total			62	592	1058	3547	7743

**Table 2:** Detection of additional AFP cases attributed to AVADAR

Country	AVADAR Districts	Priority Districts	ALERTS USING AVADAR				#AFP Via Traditional System	Total AFP (AVADAR+Trad)
			#Alerts Received	#Alerts investigated	% Alerts investigated	#of true AFP Cases		
*Chad*	9	17	15,362	1,4666	95%	118	85	203
*Niger*	6	6	1,440	1,284	89%	110	46	156
*Nigeria*	6	15	169	165	98%	89	81	170
Cameroon	4	10	3,048	2,715	89%	72	33	105
TOTAL	25	48	20,019	16,830	94%	389	245	634

## Abbreviations

CI: Community Informant
DSNO: (investigators)
AFP: Acute Flaccid Paralysis
NPAFP: non-polio acute flaccid paralysis
WPV: Wild Polio Virus
VDPV: Vaccine-derived poliovirus
cVDPV: Circulating vaccine-derived poliovirus
GPLN: Global Polio Laboratory Network
